# Is Protean Career Attitude Beneficial for Both Employees and Organizations? Investigating the Mediating Effects of Knowing Career Competencies

**DOI:** 10.3389/fpsyg.2019.01284

**Published:** 2019-06-04

**Authors:** Razia Sultana, Omer Farooq Malik

**Affiliations:** ^1^ Department of Management Sciences, COMSATS University Islamabad, Islamabad, Pakistan; ^2^ College of Business Administration, Al Yamamah University, Riyadh, Saudi Arabia

**Keywords:** protean career attitude, career insight, networking, job-related skills, career success, task performance, Pakistan

## Abstract

The aim of this study was to investigate the direct and indirect effects of protean career attitude on subjective and objective career success representing personal outcomes and task performance reflecting an organizational outcome. Drawing on the intelligent career framework, three knowing career competencies, i.e., career insight (knowing why), networking (knowing whom), and career/job-related skills (knowing how), were hypothesized as mediators linking protean career attitude with its personal and organizational outcomes. Participants of the study were 241 faculty members and matched supervisors from five large public sector universities in Islamabad, Pakistan. Data were collected in two waves through a personally administered questionnaire and analyzed through covariance-based structural equation modeling (CB-SEM). Results showed that protean career attitude has direct positive impacts on subjective career success, objective career success, and task performance. Further, the mediating role of three knowing career competencies was partially supported. We contribute to the literature by proposing and testing a research model linking protean career attitude with its personal and organizational outcomes directly and indirectly through three ways of knowing. A number of practical implications along with future research directions are also discussed.

## Introduction

The challenges of the post-1990 era (i.e., globalization, rapid technological advancements, economic recessions) have initiated a cost-efficient use of labor through layoffs, outsourcing, and contractual jobs (Hall, 2004). Consequently, employees have been induced to develop agentic career attitudes to survive in a volatile work environment (Herrmann et al., 2015). One of these attitudes is protean career attitude (PCA) which has been characterized as “involving greater mobility, a more whole-life perspective, and a developmental progression” (Briscoe et al., 2006, p. 31). Hall (1976) was one of the pioneers to recognize and respond to potential shifts in the context of individual careers. Named after Proteus, the Greek god who was able to change his shape at will, a protean career is driven by the individual, not the organization, and that will be reinvented by the individual from time to time as the individual and the environment change (Hall, 1996; Hall and Moss, 1998). Hall (1996) described psychological success of an individual in terms of his or her vision and central values and claimed that “the path to the top has been replaced by the path with a heart” (p. 10). The path with a heart is quite compatible with the notion of “following a calling” and involves one’s own most-appreciated talents, facilitates personal development, and besides benefiting the individual and his or her family, it serves the community or the larger society (Hall and Chandler, 2005). PCA is defined as “the extent to which an individual manages his or her career in a proactive, self-directed way driven by personal values and subjective success criteria” (Waters et al., 2014, p. 405). Individuals with a PCA are self-directed as they follow their careers based on personally defined career goals, and they are values-driven as they shape their career in accordance with their own internal values and beliefs rather than organizational values and beliefs (Volmer and Spurk, 2011). In other words, protean individuals are self-directed and values-driven.

Since its inception, various researchers have conducted systematic literature reviews for the operationalization and development of PCA (e.g., Sullivan and Baruch, 2009; Gubler et al., 2014). Researchers have also investigated the relationship between PCA and several personal and organizational outcomes such as career success (De Vos and Soens, 2008), job satisfaction, organizational commitment, and intentions to quit (Supeli and Creed, 2016), leadership skills (Briscoe et al., 2010), and employability (Lin, 2015). Nonetheless, considering that the first scale to measure PCA was developed recently (Briscoe et al., 2006), many relationships are still under-explored and, importantly, there are inconsistencies in findings (DiRenzo, 2010; Baruch et al., 2014). One group of researchers argues that protean individuals are highly selfish, self-centered, and do not yield favorable organizational outcomes (Briscoe and Finkelstein, 2009; Supeli and Creed, 2016). For instance, Supeli and Creed (2016) demonstrated that higher levels of protean orientation were associated with lower levels of job satisfaction and organizational commitment and higher turnover intentions. Another group of researchers claims that a PCA is beneficial for both employees and organizations (e.g., Skromme Granrose and Baccili, 2006). For instance, Sargent and Domberger (2007) in their study found that protean individuals are engaged in benefiting others. They are inclined toward learning and development and have the adaptability to embrace change which assists them in achieving organizational goals. Similarly, Waters et al. (2014) demonstrated that a PCA results in gains for both employees and organizations. Thus, PCA’s contribution in positively shaping organizational outcomes cannot be overlooked.

Researchers have examined the impact of PCA on personal and organizational outcomes, but limited studies have proposed research models which simultaneously investigate the impacts of PCA on personal and organizational outcomes. This holistic view of PCA is indispensable as it epitomizes the advantages of having a PCA for the benefit of both employees and organizations, thereby extending its scope as an integral attitude vital for the survival and long-term sustainability of an organization. A notable exception is a study conducted by Rodrigues et al. (2015) who examined the impact of PCA and boundaryless career orientation on both personal (e.g., job satisfaction, career satisfaction, and life satisfaction) and organizational outcomes (e.g., job performance, organizational citizenship behaviors, and organizational commitment). Nonetheless, PCA and boundaryless career orientation are related; yet, different constructs, and researchers, like Hall et al. (2002), have emphasized studying PCA independent of boundaryless career orientation. In the current study, we thus focus exclusively on the former and investigate its impact on two personal outcomes (i.e., subjective and objective career success) and one organizational outcome (i.e., task performance) among a sample of university faculty in Pakistan. Additionally, we attempt to examine the mediating effects of three knowing career competencies responsible for linking PCA with personal and organizational outcomes.

Previous studies have identified some mediators linking PCA with its outcomes such as career engagement (De Vos and Segers, 2013), learning-goal orientation (Lin, 2015), career self-management (De Vos and Soens, 2008), and motivational orientation (Kaspi-Baruch, 2016); however, these studies have several shortcomings. First, these studies did not propose a mediational model based on any contemporary career framework. Second, some studies have defined PCA as a multidimensional construct comprising two dimensions (i.e., self-directed and values-driven career attitude) and investigated their individual impacts on outcome variables (e.g., Enache et al., 2011; Volmer and Spurk, 2011). On the other hand, some studies have focused exclusively on either self-direction or values-driven predispositions (e.g., De Vos and Soens, 2008; Rodrigues et al., 2015). Third, these researchers did not integrate both personal and organizational outcomes in their research models, thus limiting our understanding regarding PCA’s role in benefiting both employees and organizations. To overcome these limitations, we propose a mediation mechanism that is based on the intelligent career framework (Arthur et al., 1995) with an aim to explore the black box linking PCA with its personal and organizational outcomes. The pursuit of a mediating mechanism linking PCA with its outcomes is consistent with the recent recommendations of prominent researchers in the field (e.g., Hall et al., 2018). According to Parker et al. (2009), individuals accomplish their career goals by managing three knowing career competencies, i.e., “knowing why” (“reflecting an individual’s motivation and identity”), “knowing whom” (“reflecting an individual’s relationships both inside and outside the workplace”), and “knowing how” (“reflecting an individual’s job-related skills and expertise”). Eby et al. (2003) identified several variables which represent three knowing career competencies. Following their suggestions, in our research model, career insight represents knowing why, networking reflects knowing whom, and career/job-related skills represent knowing why competencies. Specifically, we argue that a PCA along with three knowing career competencies may play a vital role in generating desired outcomes for both employees and organizations. Moreover, in the current study, we define PCA as a unidimensional construct following the suggestion of Waters et al. (2014). The union approach of PCA submits that an individual who is both self-directed and values-driven is most fully protean (Briscoe and Hall, 2006). Thus, the strongest manifestation of PCA is a combination in which an individual is high in both self-directed and values-driven career attitudes (Waters et al., 2014). Lastly, and importantly, we take a more holistic view of PCA in an attempt to gain a better understanding of its beneficial impacts on both personal and organizational outcomes.

Gubler et al. (2014) noted that research to date that has examined PCA has been conducted mainly in Western cultures, whereas little research has been carried out to explore whether the concept of PCA is applicable and generalizable in non-Western cultures, particularly those with high levels of collectivism. The individualistic values and agency inherent in PCA might not be straightforward to transfer into a collectivist culture because autonomy-related values and self-directedness have less of an expression in such cultures (Supeli and Creed, 2016). Pakistan is a developing country and its culture is known to be highly collectivist where fitting in with one’s group is an important consideration (Kashif et al., 2016). Individuals ascribing to strong collectivist values are more likely to rely on their organizations for managing their careers rather than developing a PCA and, consequently, not fully realizing its beneficial effects. Traditional employment relationships, which guaranteed job security to an employee in exchange for his or her loyalty, are becoming increasingly rare in advanced countries, and developing countries are also moving in the same direction (Sullivan, 1999). Replacing this is the new employment relationship in which the employer offers an employee a challenging job along with the promise of opportunities to learn new skills. The employee, in turn, reciprocates the employer through enhanced job performance (Tsui and Wu, 2005). Although culture is not the primary focus of this study, it is useful to explore the applicability of the PCA construct in a more collectivist culture (i.e., Pakistan) and its potential beneficial impacts on personal and organizational outcomes as it may have important practical implications for both the employee and the employer. Toward this end, we focus on the higher education sector of Pakistan, which has undergone substantial changes over the last few years. Specifically, the higher education sector has adopted and rigorously applied Western criteria for faculty hiring and firing, performance evaluations, and promotions. Consequently, these drastic changes in the employment relationships have necessitated the need for academicians in Pakistan to develop a PCA in order to attain desired career outcomes and exhibit superior performance to survive in the contemporary workplace.

In the current study, we empirically examine the direct and indirect effects of PCA on personal and organizational outcomes mediated through three knowing career competencies. Utilizing the intelligent career framework (Arthur et al., 1995), in particular, we (1) examine the direct effects of PCA on subjective career success, objective career success, and task performance and (2) investigate the mediating effects of three knowing career competencies—i.e., career insight (knowing why), networking (knowing whom), and career/job-related skills (knowing how) in the relationships between PCA and personal and organizational outcomes.

## Theoretical Background and Hypotheses Development

### Protean Career Attitude and Subjective Career Success

Career success is defined as “the positive psychological or work-related outcomes or achievements one accumulates as a result of work experiences” (Seibert et al., 1999, p. 417), which implies both subjective success and objective success. Subjective career success is defined as “an individual’s positive evaluation of his/her career” (Volmer and Spurk, 2011, p. 208). Criteria for subjective success are, for instance, career satisfaction, job satisfaction, or comparative judgments (Ng et al., 2005). Wiernik and Kostal (2019) noted that a key feature of protean individuals is a concern for psychological success, as opposed to concern only for extrinsic rewards. According to Rodrigues et al. (2015), PCA is associated with psychological and career resources such as proactivity, career adaptability, and effective coping with uncertainty, and leads to favorable personal outcomes (e.g., job satisfaction, career satisfaction, and life satisfaction). Self-determination theory (SDT; Deci and Ryan, 1985) addresses factors that either facilitate or undermine motivation, both intrinsic and extrinsic. Central to SDT is the distinction between autonomous motivation and controlled motivation (Deci and Ryan, 2008). When people engage in a task because they find it interesting, they are doing the task wholly volitionally (e.g., I work because it is enjoyable). Controlled motivation, on the other hand, involves engaging in a task with a sense of pressure, a sense of having to engage in the task (Gagné and Deci, 2005). Thus, in controlled motivation, one’s behavior is a function of external contingencies of reward or punishment (Deci and Ryan, 2008). Quigley and Tymon (2006) developed an integrated model that explained the mechanisms through which intrinsic motivation can influence career self-management and subsequent career success. The model postulates that when career self-management is an outgrowth of intrinsic motivation—comprising feelings of meaningfulness, choice, competence, and progress—with respect to one’s career, successful career self-management is likely to result in subjective career success (Quigley and Tymon, 2006). Supporting these arguments, Volmer and Spurk (2011) in their study demonstrated that self-directed career management was positively linked with career satisfaction. De Vos and Soens (2008) also reported a positive association between career self-management and subjective career success. In the same vein, Grimland et al. (2012) found a positive association between PCA and career satisfaction. Thus, we hypothesize:

**Hypothesis 1**: Protean career attitude is positively associated with subjective career success.

### Protean Career Attitude and Objective Career Success

Objective career success is defined as “directly observable, measurable, and verifiable by an impartial third party” (Heslin, 2005, p. 114). Indicators of objective career success are, for instance, pay, promotions, and occupational status (Judge et al., 1995). According to contest-mobility perspective of career success, an employee can only climb the organizational ladder on the basis of his or her own abilities and contributions. Employees compete with each other in an open and fair contest for progression, and victory comes to those who have more achievements (Ng et al., 2005). Following the contest-mobility perspective, we argue that individuals with a PCA engage in continuous learning and skill development activities relevant to work that translates into objective career success (e.g., higher salaries and promotions) as they fulfill current job requirements, actively contribute in achieving organizational objectives, and are more adaptable and optimistic in unexpected career circumstances, e.g., downsizing (Volmer and Spurk, 2011). In support of these arguments, Seibert et al. (1999) reported a positive association between proactivity (an important personality trait of protean individuals) and objective career success. Similarly, Volmer and Spurk (2011) reported a positive association between self-directed career management as a dimension of PCA and objective career success. Thus, we expect:

**Hypothesis 2**: Protean career attitude is positively associated with objective career success.

### Protean Career Attitude and Task Performance

Research on protean career orientation has generally ignored organizational outcomes. This is mainly due to the fact that PCA reflects a shift of power from organization to individual resulting in an increased threat to organizations of losing important employees or at least losing the influence to manage their careers in the way they were able to in the past (Rodrigues et al., 2015). Individual job performance, which is recognized as an important organizational outcome, is likely to be most relevant to career management (Devonish and Greenidge, 2010). It is important to distinguish between two broad dimensions of job performance, i.e., task performance and contextual performance. Task performance is defined as “the effectiveness with which incumbents perform activities that contribute to the organization’s technical core” (Borman and Motowidlo, 1997, p. 99). Contextual performance, on the other hand, includes behaviors that contribute to the maintenance and enhancement of the social-psychological work environment that facilitates task performance (Borman and Motowidlo, 1997; Schat and Frone, 2011). In the current study, we focus exclusively on the former as it is one of the most important behaviors which constitute the domain of job performance (Rotundo and Sackett, 2002). Empirical evidence regarding the influence of PCA on task performance is scarce. A notable exception is a study by Rodrigues et al. (2015) which demonstrated a positive association between PCA and supervisor-rated task performance. SDT postulates that behaviors can be characterized in terms of the degree to which they are autonomous versus controlled, with autonomous motivation tending to yield more effective performance on heuristic types of tasks (Gagné and Deci, 2005). Based on SDT (Deci and Ryan, 1985), we argue that because protean individuals take ownership of their careers, they feel autonomously motivated as they experience volition or a self-endorsement of actions they undertake (Deci and Ryan, 2008). Consequently, they perform their job tasks effectively as they are consistent with what they really want to do. This leads to the following hypothesis:

**Hypothesis 3**: Protean career attitude is positively associated with task performance.

### Mediating Role of Career Insight, Networking, and Career/Job-Related Skills

As mentioned earlier, we investigate the mediating role of three knowing career competencies derived from the intelligent career framework (Arthur et al., 1995). The “knowing why” mediating variable in the present study is career insight. Career insight is defined as “the realism and clarity of the individual’s career goals” (London, 1993, p. 55). It reflects the arousal component of career motivation and includes having self-knowledge—particularly, realizing one’s own strengths and weaknesses (Eby et al., 2003; Day and Allen, 2004). Based on the operationalization of PCA as reflecting a feeling of personal agency (Briscoe et al., 2006), it can be expected that protean individuals strive hard to develop a strong career insight as it facilitates goal clarity and its future orientation allows them to make meaningful career decisions. In turn, individuals who reflect more actively with their career goals and who have a stronger insight into what they want to achieve during their career are likely to report higher career success. In support of these arguments, De Vos and Soens (2008) in their study demonstrated that career insight mediates the relationship between PCA and career success. Moreover, it is well documented that task performance of individuals with clear, realistic, and challenging goals is superior to those who have unclear, unchallenging, or no goals (Locke et al., 1981). Taken together, we argue that protean individuals set clear and realistic career goals for themselves that, in turn, are reflected in higher levels of career success and superior task performance. This leads us to the following hypothesis:

**Hypothesis 4a**: Career insight mediates the relationships between protean career attitude and subjective career success, objective career success, and task performance.

The “knowing why” mediating variable in our research model is networking. Networking can be defined as “individuals’ attempts to develop and maintain relationships with others who have the potential to assist them in their work or career” (Forret and Dougherty, 2001, p. 284). This definition explains networking as a proactive behavior that assists in developing one’s relationship constellation with people both inside and outside their organization. Moreover, these people may have the potential to guide individuals in their work or career (Forret and Dougherty, 2004). Networking is recognized as a specific career competency which is primarily linked with the receipt of career benefits such as access to information, resources, and career sponsorship (Arthur et al., 1995; Seibert et al., 2001). Protean individuals actively pursue networking as a career management strategy because they firmly believe that their employer is no more responsible for managing their careers (Forret and Dougherty, 2004). The model of social capital and career success (Seibert et al., 2001) posits that benefits associated with networking are positively associated with both subjective and objective career success. Wolff and Moser (2009) in their study demonstrated that networking is positively related with intrinsic and extrinsic career success. In the same vein, Eby et al. (2003) demonstrated that internal and external networking is positively associated with perceived career success. Moreover, it is well documented in the literature that task performance of individuals who possess strong networking skills is better than those who possess weak networking skills (Thompson, 2005). Taken together, we argue that protean individuals demonstrate a strong preference to build networks both inside and outside the organization with the intention of gaining access to valuable social resources that, in turn, manifest in higher levels of career success and superior task performance. Thus, we hypothesize:

**Hypothesis 4b**: Networking mediates the relationships between protean career attitude and subjective career success, objective career success, and task performance.

The “knowing why” mediating variable in our research model is career/job-related skills. The emphasis of “knowing how” competency is on developing a wide and flexible skill base that is transferable across organizational boundaries (DeFillippi and Arthur, 1996). Thus, the focus is on occupational learning rather than job-related learning, which makes it unique from traditional discussions of human capital—e.g., formal education and training (Parker et al., 2009). Career/job-related skills accumulate over time and contribute to both the individual’s and organization’s knowledge base (Eby et al., 2003). Research has demonstrated that individuals who invest in constantly updating their job-related skills report higher levels of career success (e.g., Eby et al., 2003; De Vos et al., 2011). In addition, research reveals that individuals who possess a diversified set of job-related skills demonstrate higher levels of job performance (e.g., Morgeson et al., 2005). The protean career prescribed by Hall (1996) requires individuals to attain and employ an identifiable set of skills that facilitate adaptability in their working environment (Lee, 2003). We argue that individuals having a PCA engage in a lifetime of learning that, in turn, reflects in higher levels of career success and also benefits the organization in the form of greater task performance. Thus, we expect:

**Hypothesis 4c**: Career/job-related skills mediate the relationships between protean career attitude and subjective career success, objective career success, and task performance.

**Figure 1:**
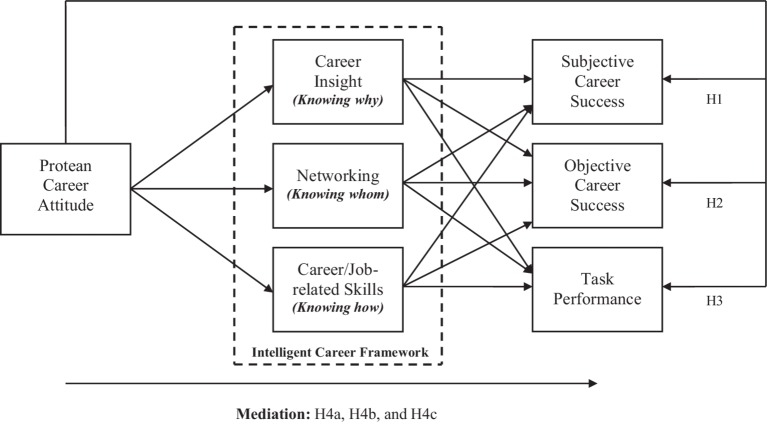
The research model.

Figure 1 depicts the research model. The model posits that three knowing competencies (i.e., career insight, networking, and career/job-related skills) derived from the intelligent career framework partially mediate the relationships between PCA and personal and organizational outcomes. We also compared the proposed partially mediated model with two alternate models.

## Materials and Methods

### Context of the Research, Participants, and Procedure

Public sector universities in Pakistan are regulated by the Higher Education Commission, Pakistan (HEC). Since its inception in 2002, the HEC has taken many initiatives to improve the quality of teaching and research in public sector universities including faculty development programs for capacity enhancement of universities. The HEC introduced the Tenure Track System (TTS) in 2005 to gradually replace the traditional Basic Pay Scales (BPS) system in public sector universities. Currently, almost all public sector universities are recruiting new faculty under the TTS and the minimum qualification for entry-level positions has been raised to a doctorate degree. Although the transition from the BPS to the TTS has promoted the research culture in Pakistani universities, it has also created challenges for the faculty members in terms of job insecurity, unusual delays in promotions, and, above all, the new system offers no early or normal retirement benefits. This transition has implicitly shifted the responsibility of managing the careers onto the shoulders of the individual, thus underscoring the need for developing a protean career orientation. The higher education sector in Pakistan is going through a very rapid transformation in order to bring it on a par with international standards; thus, in our opinion, the sample of faculty members of public sector universities in Pakistan (a non-Western culture that scores high on collectivism) was best suited to investigate the relationships between PCA, mediators, and outcome variables.

Participants of the study were full-time faculty members appointed on the TTS (i.e., assistant professor, associate professor, and professor) from various academic departments of five large public sector universities in Islamabad, and their direct supervisors who were the heads of department.

Data collection took place between April and June in 2018. Data were collected in two waves with a time lag of 3 weeks in order to minimize common method bias. In the first wave, we purposively selected 1,000 faculty members and the questionnaires were personally delivered to them. Participation in the survey was voluntary and the respondents were assured of both anonymity and confidentiality. The respondents rated their own PCA, career insight, networking, career/job-related skills, and subjective career success. Participants also reported their current monthly salary. The respondents were informed that their supervisors would be invited to provide their performance ratings. The respondents were assured that their responses would remain confidential and would not be seen by their supervisors. The 392 usable survey responses that were received either directly or *via* return envelope by the end of the first wave of survey constituted a 39.2% response rate.

Since supervisors play an important role in appraisal and reward systems, a subordinate’s job performance might be best rated by his or her supervisor (Janssen, 2001). Accordingly, in the second wave of data collection, we contacted 85 supervisors who were the departmental heads of the respondents who participated in the first wave and requested them to rate their subordinates’ job performance. The supervisor surveys contained the code for both the supervisor and the subordinate, whereas the subordinate surveys contained only the subordinate code. These codes were used to match the subordinate’s questionnaire with his or her supervisor’s questionnaire. We received useful responses from 53 direct supervisors, resulting in a 62.4% response rate. Because some supervisors declined to complete performance ratings, our final sample comprised 241 subordinate-supervisor dyads representing a ratio of 4.5:1. Of the subordinates, 64% were male. The average age was 42 years (SD = 5.69), and the average organizational tenure was 12 years. They were very well educated, with all holding a doctorate degree in their respective fields, whereas 8.7% had completed a postdoctoral fellowship. In the case of supervisors, 85% were male and all had a doctorate degree. The mean age was 44 years (SD = 5.88). Demographic characteristics of both subordinates and supervisors are summarized in Table 1.

**Table 1:** Study sample demographics.

		Subordinates (*n* = 241)	Supervisors (*n* = 53)
Gender	Male Female	64% 36%	85% 15%
Age	20–29 years old 30–39 years old 40–49 years old 50 years old or above	13.0% 43.2% 28.3% 15.5%	0% 45.2% 52.6% 2.2%
Education	A doctorate degree Postdoctoral fellowship	91.3% 8.7%	90.7% 9.3%
Organizational tenure	Less than 5 years Between 5 and 10 years More than 10 years	12.7% 63.9% 23.4%	14.3% 65.9% 19.8%
Current status	Probation Tenured	82% 18%	72% 28%

### Measures

PCA was measured through a 14-item scale developed by Briscoe et al. (2006). It consists of two subscales: self-directed career management attitude (eight items; e.g., “Ultimately, I depend on myself to move my career forward”) and a values-driven career attitude (six items; e.g., “I navigate my own career, based on my personal priorities, as oppose to my employer’s priorities”). Scale endpoints were labeled from 1 (to little or no extent) to 5 (to a great extent). Confirmatory factor analysis (CFA) demonstrated better model fit for one global PCA factor [χ^2^ (77) = 553.087, *p* < 0.001; RMSEA = 0.073; CFI = 0.850; SRMR = 0.056] compared to two separate factors of self-direction and values-driven [χ^2^ (76) = 661.081, *p* < 0.001; RMSEA = 0.084; CFI = 0.733; SRMR = 0.077]. Composite reliability (CR) was 0.941. Career insight was assessed with a five-item scale developed by London (1993). A sample item is “I have clear career goals.” Scale endpoints were labeled from 1 (strongly disagree) to 5 (strongly agree). CR was 0.902. Networking was assessed with three items adopted from Chow and Chan (2008). A sample item is “In general, I am very close to my organizational members.” Scale anchors ranged from 1 (strongly disagree) to 5 (strongly agree). CR was 0.803. Career/job-related skills were measured with five items developed by Eby et al. (2003). A sample item is “I have a diversified set of job related skills.” Scale endpoints were labeled from 1 (strongly disagree) to 5 (strongly agree). CR was 0.893. Subjective career satisfaction was operationalized in terms of career satisfaction. Career satisfaction was measured with a five-item scale developed by Greenhaus et al. (1990). Sample item includes “I am satisfied with the progress I have made toward meeting my overall career goals.” Scale endpoints were labeled from 1 (strongly disagree) to 5 (strongly agree). CR was 0.883. Objective career success was assessed by asking respondents to indicate their current monthly salary. Using salary to measure objective career success is consistent with the practice of other researchers (e.g., Seibert and Kraimer, 2001; Poon, 2004; Ballout, 2009). We transformed the salary variable using a natural logarithmic transformation (Gerhart and Milkovich, 1989). Finally, task performance was measured with a seven-item scale developed by Williams and Anderson (1991) and was completed by supervisors. A sample item is “This employee meets formal performance requirements of his or her job.” Scale endpoints were labeled from 1 (strongly disagree) to 5 (strongly agree). CR was 0.919. Age, gender, education, and tenure were control variables as they are related to the outcome variables (Rodrigues et al., 2015).

### Data Analyses Strategy

Structural equation modeling (SEM) was carried out on raw data using IBM® SPSS® Amos. Amos is powerful SEM software with graphical user interface that can create more realistic models than standard multivariate statistics or multiple regression models alone. By default, parameters in Amos are estimated by means of maximum likelihood estimation. We followed Anderson and Gerbing’s (1988) two-step approach. First, we evaluated the measurement model to ascertain whether the observed variables served as adequate indicators of the latent variables. Second, we evaluated the structural model to examine the posited nomological network among variables. To test the significance of mediation effects, we used the bootstrapping method as recommended by Preacher and Hayes (2004). Bootstrapping, as a method to test mediation effects, has been demonstrated to have a greatest statistical power to detect significant mediation processes while maintaining acceptable Type I error rates (Cheung and Lau, 2008). We used the model trimming strategy for nested model comparison to test whether the proposed partially mediated model significantly contributed to the fit of the data than simpler models. The analysis began with the proposed model as the start and simplified it with deleting paths. Specifically, two alternate models were proposed in the nested comparisons, i.e., the fully mediated model and the non-mediated model. The chi-square difference test was used to assess differences among the competing nested models. In this regard, if chi-square difference (*Δχ^2^*) is statistically significant, it implies that the more parsimonious model has a statistically significant worse fit than does the less parsimonious model (Keith, 2015).

## Results

### Common Method Bias

Common method bias (CMB) can be a serious issue when data are collected from a single source. Following the suggestions of Podsakoff et al. (2012), we assessed the prevalence of common method bias using the Harman single-factor test. Results showed that the indicators did not load significantly onto one single factor but rather six different factors. The first factor only explained 38.07% of variance in the items. Thus, CMB did not pose a serious problem with regard to collected data.

### Measurement Model

Before proceeding to the evaluation of the structural model, we ran a CFA in Amos 20 using maximum likelihood estimation. The results of CFA clearly favored a six-factor solution. However, the initial CFA model did not fit the data well: *χ^2^* (720) = 1944.531, *p* < 0.001; RMSEA = 0.084; CFI = 0.850; and SRMR = 0.063. In order to achieve better fit, two items from the PCA scale and one item from the task performance scale were removed due to low factor loadings (i.e., less than 0.7). The modified CFA model fitted the data well: *χ^2^* (607) = 1278.488, *p* < 0.001; RMSEA = 0.068; CFI = 0.907; and SRMR = 0.058. The scales were reliable as the CR score of each scale was greater than the cutoff value of 0.7, ranging from 0.803 to 0.941. Convergent validity was established as factor loadings of indicators on their respective constructs in the modified CFA model were greater than 0.7, and the average variance extracted (AVE) value of each construct exceeded the threshold value of 0.5, ranging from 0.578 to 0.661 (Hair et al., 2018; Ringle et al., 2018). Discriminant validity was established using the Fornell and Larcker (1981) criterion. As shown in Table 2, for each scale, the square root of its AVE estimate was greater than the construct’s respective correlation with all constructs. Taken together, the psychometric properties of all scales were satisfactory.

**Table 2:** Means, standard deviations, and correlations among constructs.

Construct	Mean	SD	1	2	3	4	5	6	7
Protean career attitude	3.981	0.599	**0.813**						
Career insight	3.979	0.579	0.335	**0.808**					
Networking	3.998	0.408	0.504	0.322	**0.760**				
Career/job-related skills	3.715	0.672	0.405	0.386	0.385	**0.792**			
Subjective career success	3.777	0.563	0.495	0.343	0.266	0.315	**0.777**		
Objective career success^a^	10.710	2.005	0.474	0.263	0.514	0.297	0.276	-	
Task performance	4.049	0.562	0.441	0.447	0.380	0.532	0.290	0.227	**0.811**

*Square root of the average variance extracted value on the diagonal*.

^a^*Natural logarithm; All correlations are significant at p < 0.01*.

### Structural Model

**Figure 2:**
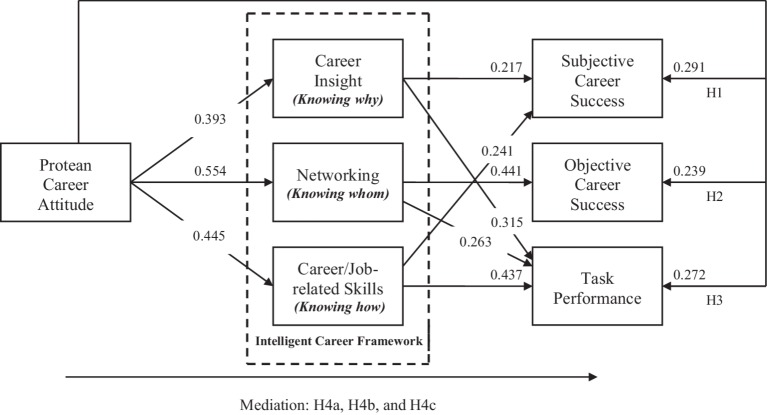
Path coefficients are from the completely standardized solution and are significant at *p* < 0.05.

As mentioned earlier, we compared the proposed partially mediated model (Model 1) with two competing models—the fully mediated model (Model 2) and the non-mediated model (Model 3). The fully mediated model was created in which the paths linking PCA with outcome variables were fixed to be zero. On the other hand, a non-mediated model was created in which the paths linking three knowing career competencies (i.e., career insight, networking, and career/job-related skills) with outcomes variables were fixed to be zero. As the results shown in Table 3 indicate, the partially mediated model (Model 1) provided the best fit for the data. Since Model 2 and Model 3 are nested within Model 1, a change in chi-square statistic was performed between Model 2 and Model 1, and Model 3 and Model 1. The chi-square difference between Model 2 and Model 1 was 48.852 with three degrees of freedom (*p* < 0.001), and the chi-square difference between Model 3 and 1 was 100.387 with six degrees of freedom (*p* < 0.001). Overall, Model 1, the partially mediated model of the intelligent career framework, appeared to provide a superior fit for the data as compared with Model 2 and Model 3. Figure 2 shows the standardized path coefficients for the partially mediated model. As expected, PCA was positively and significantly related to subjective career success (*β =* 0.291; *t* = 4.662), objective career success (*β =* 0.239; *t* = 4.045), and task performance (*β =* 0.272; *t* = 3.377). Together, these results (summarized in Table 4) lent support to Hypotheses 1, 2, and 3. None of the control variables were found to be significantly related with the outcome variables.

**Table 3:** Fit indices of tested structural models.

Hypothesized model	χ^2^	*df*	CFI	RMSEA	SRMR
Model 1 (partially mediated)	1313.410	616	0.903	0.069	0.089
Model 2 (fully mediated)	1362.262	619	0.897	0.071	0.140
Model 3 (non-mediated)	1413.797	622	0.890	0.073	0.104

**Table 4:** Direct effects of PCA on outcome variables.

Path	Coefficient	*t*	95% Bias-corrected bootstrap confidence interval
PCA → subjective career success	0.291	4.662	(0.273, 0.304)
PCA → objective career success	0.239	4.045	(0.211, 0.251)
PCA → task performance	0.272	3.377	(0.251, 0.293)

To assess the significance of specific indirect effects, we used the phantom model approach (Macho and Ledermann, 2011). The indirect effect is statistically significant if zero is not between the lower and upper bound of the confidence interval (Hayes, 2009). The results of the phantom model analysis using 1,000 bootstrap samples revealed that career insight mediates the impact of PCA on subjective career success and task performance with point estimates and 95% bias-corrected bootstrap confidence intervals of 0.086 (0.032, 0.169) and 0.129 (0.081, 0.211), respectively, but did not mediate the effect of PCA on objective career success. Thus, Hypothesis 4a was partially supported. Further, the results showed that networking mediates the effects of PCA on objective career success and task performance with point estimates and 95% bias-corrected bootstrap confidence intervals of 0.249 (0.114, 0.311) and 0.149 (0.071, 0.221), respectively, but did not mediate the relationship between PCA and subjective career success. Hence, Hypothesis 4b was also partially supported. Finally, the results revealed that career/job-related skills mediate the effects of PCA on subjective career success and task performance, with point estimates and 95% bias-corrected bootstrap confidence intervals of 0.109 (0.069, 0.188) and 0.197 (0.091, 0.251), respectively, but did not mediate the relationship between PCA and objective career success, thereby providing partial support for Hypothesis 4c.

## Discussion

The main purpose of this study was to investigate the direct and indirect effects of PCA on personal and organizational outcomes mediated through three ways of knowing (i.e., career insight, networking, and career/job-related skills) drawn from the intelligent career framework. We found that PCA is relatively normally distributed (skewness statistic = −0.482, SE = 0.157; kurtosis statistic = 0.794, SE = 0.312), implying that, in this sample of faculty members from a non-Western culture high on collectivism, the said attitude is distributed in nearly similar fashion as found in studies comprising samples from Western cultures (e.g., Waters et al., 2014), as well as other non-Western cultures high on collectivism (e.g., Supeli and Creed, 2016). Results showed that higher levels of PCA are positively associated with personal outcomes of subjective and objective career success and task performance representing an organizational outcome. These findings are consistent with previous research suggesting that protean career individuals not only benefit themselves by achieving higher levels of intrinsic and extrinsic career success, but also the organization in terms of exhibiting superior job performance (Rodrigues et al., 2015). The results are also in line with King’s (2004), p. 122–123, assertion that “through successful use of career self-managing behaviors over a sustained period, people master their development tasks and, as a result, achieve their desired career outcomes.” The results of the mediation analyses indicated that career insight representing the “knowing why” career competency partially mediates the impacts of PCA on subjective career success and task performance. However, career insight did not mediate the impact of PCA on objective career success and was not significantly related to the latter. The findings suggest that protean individuals are characterized to set realistic, challenging, and attainable career goals for themselves which, in turn, leads to higher levels of intrinsic career success and greater task performance due to increased intrinsic interest in job activities (Deci and Ryan, 2008). However, a stronger career insight may not necessarily be reflected in higher levels of extrinsic career success for protean individuals. The results further revealed that networking, representing the “knowing whom” career competency, partially mediates the effects of PCA on objective career success and task performance, but did not mediate the relationship between PCA and subjective career success. The findings suggest that protean individuals exhibit a great tendency to actively engage in building networks with people both inside and outside their organizations, and as a result they are likely to gain access to information and resources (Seibert et al., 2001). Consequently, gaining sensitive and detailed information from one’s opponent leads to increased negotiation performance such as obtaining the desired salary level (Seidel et al., 2000). In addition, network building may embolden individuals to exercise discretion in pursuing initiatives that go beyond their formal job duties besides providing increased opportunity to attach themselves to people who occupy positions of power and influence in the organization that could to lead to heightened performance evaluations (Thompson, 2005). Finally, the results demonstrated that career/job-related skills representing “knowing how” career competency partially mediate the effects of PCA on subjective career success and task performance but did not mediate the effect of PCA on objective career success. These findings suggest that protean individuals are inclined to seek out opportunities for continuous learning, which makes their careers more intrinsically satisfying because they have a strong sense of professional identity (Eby et al., 2003). Additionally, individuals who constantly update their job-related skills are likely to broaden their roles, leading to higher ratings of individual job performance (Morgeson et al., 2005). Contrary to our expectations, however, we found that individuals who possess a diversified set of job-related skills may not necessarily achieve extrinsic career progression. Taken together, the results of the mediation analyses demonstrated that three knowing career competencies differentially relate PCA with outcome variables. Importantly, all three knowing career competencies, i.e., career insight, networking, and career/job-related skills mediated the relationships between PCA and task performance, thus underscoring the importance that a protean individual must strive to acquire these three knowing career competencies in order to perform their job tasks effectively. Consequently, it can be argued that protean individuals who have a strong career insight, actively engage in network building, and posses a diversified set of job-related skills are most beneficial for their organizations.

It should be noted that our results are not entirely consistent with previous studies that have examined the impacts of PCA on personal and organizational outcomes in the context of developing countries which score high on collectivism. For example, Supeli and Creed (2016) surveyed young adults who worked for a large electronics manufacturing company in Indonesia and reported that higher levels of protean career orientation were associated with lower levels of organizational commitment and job satisfaction and higher levels of intentions to quit, indicating that protean individuals do not benefit themselves or their organizations. The differences in results may have two plausible explanations. First, in the current study, we surveyed highly qualified university faculty who frequently use online communication technologies and often travel around the world, particularly Western countries, to attend conferences, seminars, and workshops and, as a result, they are more likely to adopt Western attitudes (e.g., PCA). Second, since the public sector universities in Pakistan have adopted the TTS for faculty appointments—the concept that originated in the United States—it is likely that these institutions over time have become alerted to meeting the needs of employees who have more individualistic orientations and have provided them the opportunity to operationalize their values in the workplace, ultimately leading to mutual gains as demonstrated in the current study. Thus, it can be argued that the concept of PCA is tenable in certain work contexts, but not in others, in cultures high on collectivism.

### Theoretical Implications

The study has several theoretical implications. First, we follow the union approach to PCA, which suggests that the strongest demonstration of PCA is a combination in which an individual is high in both self-directed and values-driven career attitudes (e.g., Briscoe and Hall, 2006; Waters et al., 2014). The results of CFA showed that self-directed and values-driven career attitudes both load onto a global PCA factor. Therefore, our results lend support for the unidimensionality of the PCA scale in a non-Western culture that scores high on collectivism (i.e., Pakistan). Second, we took a more holistic view of PCA by testing a research model that proposed the impacts of PCA on both personal and organizational outcomes. The rationale for incorporating both outcomes in the model was based on the fact that the majority of the studies linking PCA with its outcomes have focused exclusively on personal outcomes to the exclusion of organizational outcomes, and a few studies which have included both outcomes have yielded inconsistent results (e.g., Rodrigues et al., 2015; Supeli and Creed, 2016). Third, to the best of our knowledge, this is the first study to utilize the intelligent career framework as a mediation mechanism linking PCA with its personal and organizational outcomes. Specifically, we demonstrated that three knowing career competencies (i.e., why, whom, and how) play a vital role in transmitting the impacts of PCA on outcome variables. Lastly, following the recommendations of Hall et al. (2018), we have attempted to broaden the nomological network of PCA by including constructs in our model which are responsible for transmitting the beneficial effects of PCA on important personal and organizational outcomes.

### Practical Implications

The study offers several practical implications for both individuals and organizations. First, in an age of changing employment relationships, employees should understand the importance of self-managing their careers driven by their own personal values and how such a career management initiative may help them in achieving higher levels of career success (both intrinsic and extrinsic) and exhibiting superior job performance, thus benefiting themselves and their organizations. However, employees in pursuit of self-managing their careers should fully realize the importance of having a strong career insight, building networks with people both inside and outside their organization, and engaging in continuous learning activities for achieving desired career outcomes and reciprocating the organization through superior job performance (Eby et al., 2003). Second, organizations should also encourage their employees to take charge of their own careers and facilitate them in developing the required career competencies. In this regard, organizations can initiate career counseling programs which can assist the employees in identifying specific career goals and knowing their strengths and weaknesses. Organizations should also provide their employees the opportunity to construct networks with people both inside and outside the organization. In the context of higher educational institutions, networking opportunities can be provided to employees by organizing conferences, seminars, and workshops in which key academic, government, and business personnel are invited. It is also the responsibility of employees to fully participate in such professional activities as well as in company-sponsored social events in order to enhance their social networks. Employees are also advised to increase their internal visibility by accepting new and challenging work assignments (Forret and Dougherty, 2001). Moreover, organizations should provide their employees the opportunity for continuous learning and development. In higher education institutions, these opportunities can be provided by awarding research grants and scholarships for higher education including postdoctoral studies. Lastly, and importantly, organizations in which traditional career paths are increasingly becoming rare should promote and reward protean individuals (Hall et al., 2018).

### Limitations and Future Research Directions

The study has some limitations that provide opportunities for future research. First, we used a cross-sectional design. Longitudinal research is warranted to come closer to causality inferences on the relationships examined in the current study. However, evidence of reverse or reciprocal causality between PCA and personal and organizational outcomes is weak or nonexistent (e.g., Supeli and Creed, 2016). Second, although we identified variables from the literature representing three knowing career competencies, we did not examine the interrelationships between these variables. For example, it can be argued that individuals who actively engage in networking behaviors may get attached to a mentor who can help them acquire new skills, knowledge, and abilities (i.e., knowing whom to knowing how). Thus, future research may examine the interplay among three ways of knowing as suggested by Parker et al. (2009). Moreover, future research may investigate the mediating role of other variables that represent knowing career competencies, but have received less research attention (e.g., openness to experience as a “knowing why” competency; Eby et al., 2003). Third, we only investigated the impact of PCA on one organizational outcome i.e., task performance. Future research may consider several other important organizational outcomes (e.g., organizational commitment, organizational citizenship behaviors, and work engagement). Fourth, data were only collected from faculty members of public sector universities in Pakistan, which limits the generalizability of the results. It should be noted that the current sample was comprised of highly educated individuals, which may have resulted in a greater understanding and expression of PCA among these individuals as indicated by higher scores on the construct. More research employing different samples, both within Pakistan and outside, is warranted in order to ascertain whether the results presented in this study are replicable. Lastly, we used purposive sampling (a non-probability technique) to select the study sample because no sampling frame was available listing the number of faculty members in public sector universities in Islamabad. The selection bias inherent in most non-probability sampling techniques may limit the generalizability of the results.

## Conclusion

In conclusion, we demonstrated that individuals who take the responsibility of managing their careers driven by their own intrinsic values can benefit themselves and their organizations. However, the mutual benefits can only materialize fully when protean individuals strive hard to acquire three knowing career competencies (i.e., why, whom, and how) with support from their organizations. In an era when traditional employment relationships are in decline, organizations should design and develop cultures which include elements that support and reward protean individuals.

## Ethics Statement

The authors of this article confirm that review and approval of the study were not required from any ethics committee according to the local and national guidelines. We also confirm that written informed consent was obtained from all the participants. Further, participation in the survey was voluntary and the participants were assured of both anonymity and confidentiality. We respect the codes and practices and ethics in research and, in particular, the Declaration of Helsinki.

## Author Contributions

RS is a PhD scholar working under the supervision of OM. RS and OM conceived the idea for the study and developed the research model and hypotheses. The literature review was conducted by RS. Data were collected and analyzed by RS under the guidance of OM. RS prepared the manuscript draft which was thoroughly examined by OM.

### Conflict of Interest Statement

The authors declare that the research was conducted in the absence of any commercial or financial relationships that could be construed as a potential conflict of interest.
